# Barriers and Facilitators for Participation in Brain Magnetic Resonance Imaging (MRI) Scans in Cancer Research: A Feasibility and Acceptability Analysis

**DOI:** 10.21203/rs.3.rs-4595719/v1

**Published:** 2024-07-18

**Authors:** Thushini Manuweera, Keerthana Karunakaran, Camille Baechler, Javier Rosales, Amber S. Kleckner, Paula Rosenblatt, Aaron Ciner, Ian R. Kleckner

**Affiliations:** University of Maryland, Baltimore; Massachusetts General Hospital; University of Maryland, Baltimore; University of Maryland, Baltimore; University of Maryland, Baltimore; University of Maryland, Baltimore; University of Maryland, Baltimore; University of Maryland, Baltimore

**Keywords:** brain, fMRI, equity, acceptability, facilitators, barriers

## Abstract

**Purpose:**

A growing body of research suggests that the brain is implicated in cognitive impairment, fatigue, neuropathy, pain, nausea, sleep disturbances, distress, and other prevalent and burdensome symptoms of cancer and its treatments. Despite anecdotal evidence of difficulties using gold-standard magnetic resonance imaging (MRI) to study the brain, no studies have systematically reported reasons that patients with cancer do or do not complete research MRI scans, making it difficult to understand the role of the brain related to these symptoms. The goal of this study was to investigate these reasons and to suggest possible solutions.

**Methods:**

We analyzed data from 72 patients with cancer (mostly breast and gastrointestinal) from 3 studies: MRI was mandatory in Study 1; MRI was optional in Studies 2–3. Patients provided reasons for completing or not completing optional research MRI scans.

**Results:**

The percentage of scans completed when MRI was mandatory was 76%, and when optional, it was 36%. The most common reasons for not completing optional scans were claustrophobia (40%), safety contraindications (11%), discomfort (5%), a busy MRI schedule (5%), and the scanner being too far away (4%). Older patients were more likely to complete at least one scan (log(odds) = 0.09/year, p = 0.02).

**Conclusion:**

Although brain MRI is feasible for many patients with cancer, it can be difficult or not feasible for patients with claustrophobia, safety issues, busy schedules, or transportation issues. Improving communication, comfort, and access to a scanner may help. Reducing inequities related to study participation can improve research supportive care research.

## Introduction

To meet supportive care needs of patients with cancer, healthcare providers need better methods to identify patient needs based on determinants such as cancer prognosis, pathophysiology, comorbidities, pre-existing health conditions, and symptoms from cancer and its treatment. A rapidly growing body of research suggests that the brain plays a key role in some of the most prevalent and burdensome symptoms of cancer and its treatment such as cognitive impairment [1–3], fatigue [4], neuropathy [5], pain [6], nausea and vomiting [7], sleep disturbances [8], distress [9], tissue wasting, and appetite loss [10]. Our work in chemotherapy-induced peripheral neurotoxicity (CIPN) [5, 11, 12] suggests that understanding the brain could help better prevent, treat, predict, and describe CIPN symptoms [5].

In fact, brain-based treatments may open a promising avenue of treatment approaches and prophylactics of cancer-related symptoms, which, when paired with traditional therapies, could provide a comprehensive approach to improving cancer patient’s care and overall quality of life. Preliminary evidence suggests that symptoms of CIPN may be partly alleviated by learning to modulate brain activity through non-invasive neurofeedback using electroencephalography (EEG) [13–15] as well as non-invasive repetitive transcranial magnetic stimulation treatment applied to the motor cortex of the brain [16, 17]. Cranial Electrical Stimulation, another non-invasive neuromodulation technique, has been shown to improve pain in people with advanced cancer [18]. There is strong evidence showing that patients with treatment-resistant depression can be successfully treated using deep-brain stimulation [19, 20]. Moreover, deep brain stimulation has been used to alleviate non-cancer chronic pain [21]. Yet, there is a lack of brain-based studies that focus on understanding and addressing the underlying mechanisms of symptoms and developing personalized supportive care interventions for individuals with cancer.

Most brain imaging research in cancer-related symptoms has been conducted using MRI, which is considered the gold standard of brain imaging technologies given its high spatial resolution, complete brain coverage, and non-invasive nature. However, one reason why MRI-based treatment methods have not been widely used in supportive care research in cancer is because it is thought to be difficult to implement for researchers (access, cost, expertise) and barriers faced by patients, which is our focus here. Indeed, factors such as claustrophobia, inability to find MRI scanner schedule availability during times that are convenient for the patient, and MRI safety contraindications such as metal in the body are the common barriers in MRI research. We believe that higher levels of distress (including anxiety, depression, physical discomfort etc.) and economic hardship can also result in the exclusion of patients, creating research inequities. However, despite anecdotal evidence, to our knowledge, no studies have systematically reported reasons that patients with cancer choose to complete or not complete research MRI scans.

The goal of this study was to investigate patient-level barriers and facilitators to completing MRI scans by asking patients why they did or did not choose to complete optional brain MRI scans in our studies. We hypothesized that claustrophobia and distress would be the main reasons for not completing optional MRI scans. We suggest how to address these issues to improve research equity and the development of better brain-based biomarkers that are more applicable in a clinical setting to help the greatest number of patients.

## Methods

### Participants and recruitment.

This paper reports on 3 behavioral interventional studies designed to assess the feasibility or preliminary efficacy of exercise vs. a behavioral placebo control on CIPN and the interoceptive brain system [22]. A total of N = 72 participants from the 3 studies with 2 possible MRI scans each were included and analyzed for this retrospective study. Out of these people, 48 participants did not complete at least one scan and 42 participants did complete at least one of the two MRI scanning sessions.

In **Study 1**, MRI was mandatory. Study 1 was a Phase I/II pilot feasibility randomized controlled trial (RCT) (NCT03021174) conducted at the University of Rochester Medical Center in Rochester, NY, USA from 2017–2018 (ethical approval #RSRB 66046) [12]. A total of 19 patients scheduled to receive neurotoxic chemotherapy (taxanes, platinum, vinca alkaloids, thalidomide, or bortezomib) and were deemed safe for brain MRI were included in this study.

In **Studies 2 and 3**, MRI was optional. Study 2 is a Phase II pilot feasibility randomized controlled trial (NCT03858153) initially conducted at the University of Rochester Medical Center in Rochester, NY, USA from 2019–2021 (ethical approval #RSRB 3387) then moved partway through accruals to University of Maryland Baltimore in 2021 (ethical approval #HP-0098661). A total of 39 participants with breast cancer scheduled to receive a taxane-based chemotherapy regimen were included in this study. Study 3 is a Phase II pilot feasibility randomized controlled trial (NCT05452902) conducted at the University of Maryland Baltimore starting 2022 (ethical approval #HP-00100000). A total of 14 participants with gastrointestinal cancer scheduled to receive platinum-based chemotherapy without concurrent radiation therapy were included in this study. Studies 2 and 3 were planned to have approximately half of the participants complete the scans due to budget and feasibility of recruitment. Both studies are currently ongoing.

### Eligibility.

For all studies, participants were ≥ 18 years of age, had cancer, had at least six months life expectancy, and were scheduled to receive neurotoxic chemotherapy. As a requirement to test the study intervention, all participants were safe to exercise and were not exercising more than two days per week. Study 2 required a diagnosis of breast cancer and being scheduled to receive taxane chemotherapy. Study 3 required a diagnosis of gastrointestinal cancer and being scheduled to receive platinum chemotherapy. Patients were not eligible if they were receiving surgery or radiation therapy for their cancer during the 12-week study period or were already regularly exercising. In addition, MRI contraindications were an exclusionary criterion for Study 1.

### Procedures.

During informed consent, participants were told that MRI was part of the study (Study 1) or was optional (Studies 2 and 3). Participants who were willing to try MRI were screened for MRI safety and were scheduled for a scan based on the availability of both the scanner and the participant. Participants were encouraged to try MRI scanning if they were inclined to do so and were assured that they could exit the scanner early if needed (e.g., due to discomfort, boredom, fatigue). The scan included a T1 structural scan, two resting state functional scans, a T2* weighted functional scan that tested bodily attention processing [11], and a diffusion-weighted scan. The appointment took about 90 minutes to complete (60 min in the scanner) and included an extra $100 in compensation with pictures of their brain scan for personal record (not a medical diagnosis). There was no cost to participants except their time; and no benefits beyond feelings of satisfaction from contributing to research. Participants were informed that the collected scans would not be used to diagnose or detect any abnormalities, however, any incidental findings would be read by a neuro-radiologist and the team would contact the subject’s treating oncologist. Transportation support was offered for participants in Studies 2 and 3 (car concierge service; Lyft) and free parking for all 3 studies.

### Data collection.

For the studies with optional MRI (Studies 2 and 3), we asked participants who declined MRI why they did not wish to complete the MRI scans. For participants who did complete the MRI scan, we asked why they completed the optional MRI scans. We also obtained demographic information including age, sex, race/ethnicity, education level, and cancer type.

### Analysis.

We used descriptive statistics and explored demographic predictors of whether participants completed at least one scan or declined at least one scan using multivariable logistic regression—we explored age (continuous), race (White vs. non-White), sex (female vs. not), and education level (completed at least college vs. not). Four authors (TM, IRK, CB, and JR) identified common themes describing the reasons to or not to complete the scans based on the qualitative feedback.

### Missing data.

We excluded participants who did not report reasons for completing or declining MRI (we added these questions later in the studies). We also excluded 20 scans from participants who either stopped participating after completing one MRI scan, were not asked the reason for not completing the second scan, or were yet to be offered their second scan. Although these scans were excluded from the analysis, these participants were still included in this manuscript because they provided at least one scan. After removing scans, we were left with a total of 37 scans for Study 1, and a total of 86 scans for Study 2 and 3.

## Results

The demographic information of all participants is listed in Table 1.

### Study 1: MRI mandatory.

There were 9 out of 37 (24%) instances where the MRI scan was not completed for the following reasons: the patient experienced unexpected claustrophobia (2), the MRI scanner was busy (2), the participant was identified as not MRI-safe right before the scan started (2), the participant was sick (2), and the MRI scanner was undergoing maintenance (1).

### Studies 2–3: MRI optional.

**Figure 1:**
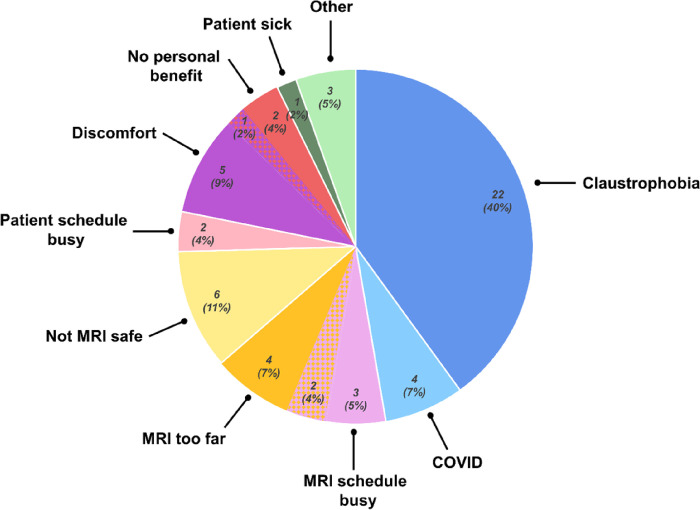
Pie chart depicting reasons for not completing optional MRI scans as reported by participants. Regions with checkered pattern depict overlapping categories.

There were 55 out of 86 (64%) instances where scanning was declined or not completed, with the following reasons provided: the patient was claustrophobic (22), the patient felt uncomfortable in the scanner (5), the MRI scanner was too far from where they lived (4), the MRI scanner schedule was busy during times that are convenient for the patient (3), the participant’s schedule was busy (2), the participant declined because there was no benefit from the scan (2), the participant was sick (1), and the participant had a family emergency (1). Some of the participants gave more than one reason for not completing the scan: the MRI scanner was too far and the MRI scanner schedule was busy (2), no benefit to the participant and discomfort (1). There were 7% of scans that were not collected due to the shutdown during COVID. These results are summarized in Fig. 1.

When MRI was optional, out of the 55 instances where the scan was not completed, 27 (49%) included reasons other than claustrophobia and MRI safety, which were exclusionary criteria for participation in the MRI mandatory (Study 1).

Younger and more educated participants were more likely to decline at least one scan (odds of declining decreases by 38%/year, p = 0.027; odds ratio for declining is 6.95 for completing college vs. not, p = 0.043), whereas sex and race were not predictive (p > 0.407). Older participants were more likely to complete at least one scan (odds of completing increases by 9%/year, p = 0.023), whereas sex, race, and education were not predictive (p > 0.172).

### Participant interview results.

We identified four common themes as to why participants declined optional MRI scanning (Table 2): the MRI is too uncomfortable (claustrophobia, scan duration), generally feeling overwhelmed, scheduling (not enough time, cannot find an appropriate time), and there was no health benefit to the participant. We also identified themes as to why participants did complete optional MRI scanning (Table 3): wanting to help future patients and contributing to research, general curiosity, and compensation. A few patients were under the false impression that the MRI scan may also benefit them since it was part of the intervention study even though prior to consent, all participants were informed that the scans did not confer any direct benefits to them.

## Discussion

Our results suggest several reasons why participants decline optional brain MRI scans. While claustrophobia, MRI safety, busy MRI scanner, and discomfort were the main reasons for not completing the MRI scan, MRI being too far, sickness, and no perceived benefit from the scan were among the other reasons. Participants reported wanting to help future patients, contributing to research, and general curiosity as reasons to complete the scans. Younger and more educated participants were more likely to decline at least one scan, whereas older participants were more likely to complete at least one scan. To our knowledge, this is the first study to discuss barriers and facilitators that patients with cancer face when choosing to complete optional brain MRI scans and to offer suggestions that may help improve study participation.

### Our results align with previous findings about barriers faced by patients undergoing MRI more generally.

While few studies were looking at barriers to MRI study participation in cancer research, some studies discussed ways to improve the MRI scan experience. Similar to our studies, key challenges were enclosed space and scan duration; but unlike our studies, participants in prior studies also reported scanner noise as a barrier; all of which were related to claustrophobia, pulmonary symptoms, and other existing comorbidities [23]. Other non-cancer related studies in neuropathic pain [24] and autism [25] reporting on participants’ acceptability of MRI scans reported barriers such as noise and scan duration, and also mentioned the importance of accessible communication and pre-scan familiarization of the MRI scanner and what to expect during the scan.

### Although mandatory MRI scans are feasible in cancer research, it may create research limitations.

Our results suggest that when the MRI scanning is mandatory the study is still feasible, given that only 24% of required scans were not completed. This 24% missing data is similar to the amount of missing data for other outcomes measured in supportive care in cancer such as daily diaries (20% missing) [26], actigraphy (22% missing), and overall study retention (26% attrition across 18 studies) [27]. However, for behavioral interventional studies that may benefit patients, such as all three studies described here, mandating MRI scanning may inadvertently introduce research inequities by excluding patients who are claustrophobic or not safe for MRI. For example, given that cancer and its treatment cause anxiety [28], patients with cancer may be more likely to experience claustrophobia [29] preventing those patients from doing a brain MRI scan. It is important that behavioral interventional studies targeted at improving supportive care include these patients, as these interventions may show beneficial effects in reducing symptoms such as anxiety [30]. Moreover, additional MRI-related study exclusions can lead to slower progress in study recruitment, especially if such studies are conducted in rural areas, which tend to be further from MRI scanners [31].

### The participant’s perceptions and understandings of research studies may influence study participation.

Studies have suggested that willingness to undergo MRI can be improved by an individual’s level of health literacy, thoroughness of education provided regarding the procedure and its purpose, and the interpersonal relationships developed with study coordinators [32]. These subjective insights align closely with existing literature on research participation more broadly (not just MRI). Factors such as higher levels of health literacy, being younger, female, or having more education are positively associated with interest and participation in clinical research [33, 34]. Conversely, low health literacy is a barrier to participation in research and optional aspects of a study such as an MRI scan. However, our data suggest that younger and more educated participants were more likely to decline scanning, perhaps due to busier lifestyles and career responsibilities, whereas older participants were more likely to be retired.

### Our study suggests ways to improve participation in brain imaging research in cancer.

As our results suggest, most patients do not complete the MRI scans because of anxiety, discomfort, a busy MRI schedule, inability to travel to the MRI scanner, and MRI contraindications. Here, we offer possible solutions to these barriers (Table 4). 1) Improve communication about the study, the MRI, the procedures, what to expect during the scan, and how these measures contribute to providing better supportive care. Educating patients regarding MRI procedures and their purpose may improve study participation by reducing anxiety, promoting acceptability, and increasing willingness to help other patients. Study participants who feel that they have been informed about MRI report higher levels of acceptability, satisfaction, and utility of MRI [33]. Indeed, educational materials and other communication, presented at the 6th-grade reading level, have been found to improve comprehension, anxiety, and levels of satisfaction [34–36]. Furthermore, providing patients with information about MRI data’s purpose to research itself further encourages participation by appealing to participants’ curiosity and desire to contribute to high-quality outcomes. Although the risk associated with participating in an MRI study is often well-communicated to participants, it is also important to clarify the benefits of the MRI to the participant (if any), as well as how it contributes to helping future patients. 2) Provide assistance and incentives to travel to the MRI scanner. 3) Ensure that the population and study time points do not exclude people unnecessarily. For example, patients with breast cancer often undergo breast reconstruction surgeries that require implanting a chest expander, which is not MRI-safe. 4) In instances described above, explore other comparable but better-suited alternative and/or portable options instead of MRI, such as functional near-infrared spectroscopy (fNIRS) or EEG.

### Even though MRI is the gold-standard for brain imaging in research, when possible, considering other brain imaging modalities such as EEG or fNIRS can help improve feasibility, research inclusivity, and accessibility in cancer studies.

The relatively mobile setup of these techniques may alleviate the anxiety and claustrophobia experienced during MRI scanning. Moreover, unlike in MRI, the use of fNIRS and EEG does not have any known safety concerns. fNIRS is an optical brain imaging technique that non-invasively quantifies brain activity via changes in blood oxygenation (similar to fMRI’s BOLD signal) [35, 36]. It is a widely used alternative to fMRI in various domains, including pain [37], pediatrics [38], and cognitive research [39] due to its superior cost, acceptability, and portability. fNIRS has been brought to patients in clinics [40, 41] and rural areas [42], and used during walking [43]. These types of imaging modalities may have a future alongside routine clinical assessment (like echocardiogram or ultrasound) to formulate brain-based treatments, risk prediction of symptoms, or their chronicity and inform treatment decisions (e.g., whether to dose-reduce chemotherapy to avoid chronic CIPN). Although fNIRS or EEG may be a good alternative to fMRI in cancer supportive care research, depending on the research question, a pilot MRI study may be necessary to determine the brain areas of interest, as these imaging techniques may be most useful in investigating the superficial cortical areas accessible by fNIRS and EEG.

### Strengths.

Our analysis provides a better understanding of the barriers and facilitators patients with cancer face when participating in an MRI study. These findings are noteworthy as our study had a moderate sample size (N = 72) and used both descriptive and quantitative methods to understand the impact of patient-related barriers on MRI research studies. We have explored these effects based on study design, mandatory MRI vs. optional MRI, and have asked participants reasons for or for not completing the MRI scan. In addition, our manuscript provides concrete action items to address barriers reported by patients, that may be helpful for other researchers interested in conducting an MRI study in patients with cancer.

### Limitations.

First, there are missing data specifying the reason for not completing the MRI scan because these questions were only added later into data collection. However, our moderate sample size was sufficient to gain a reasonable understanding of the common barriers to completing an MRI scan. Moreover, although a larger sample size might have shown additional barriers, they are likely to be less common (e.g., scanner noise, which we did not observe here, or other personal responsibilities). Second, the barriers discussed in this paper are more applicable to patients in urban areas in the US, with better access to an MRI scanning facility and potentially more likely to have better research and/or medical literacy. Conducting similar MRI studies in more rural areas may present researchers with more or different barriers in terms of study participation as well as available MRI scanner technological capabilities.

### Future work.

We believe that although having an MRI as an optional component of our study leads to more participants declining the scan, studies can be designed to include MRI as long as the sample size accounts for an estimate of what percentage of individuals will complete optional MRI. While we suggest strategies for future studies (Table 4), we will also explore quantitative analysis that will be important to better predict the completion of the MRI scans based on factors such as pre-existing conditions, level of education or research literacy, age, other participant obligations, geographic location (rural vs. suburban), and psychological wellbeing.

## Conclusion

In conclusion, MRI plays an important role in understanding the role of the brain in supportive care in cancer research and, although brain MRI studies are feasible, certain barriers faced by patients can create research inequities and setbacks in recruitment. Key barriers we observed include claustrophobia, MRI safety, busy MRI scanner schedule, discomfort, younger age, and higher education level, whereas key facilitators to completing optional scans included wanting to help future patients, contributing to research, general curiosity, and older age. When given the choice to complete optional MRI scans, many patients with cancer will do the scan and many will decline. Systematically identifying and addressing barriers and enhancing facilitators for MRI participation can enhance research conduct, improve research equity, and improve future standard of care.

## Figures and Tables

**Table 1:** Demographics and clinical characteristics of study participants.

Characteristic	Mandatory MRI study (Study 1)	Optional MRI study (Study 2 & 3)
**Total participants**	19	53
**Female sex**	10	44
**Age, years (mean ± SD)**	63.79 ± 10.95	55.23 ± 12.61
**Race**		
White	19	34
Non-White	0	19
**Education**		
At least some college	1	6
College degree	4	18
High school/GED degree	3	4
Graduate degree	3	14
**Cancer type**		
Bladder	1	0
Breast	8	39
Colon	1	2
Esophageal	1	0
Gastrointestinal	1	1
Myeloma	3	0
Pancreatic	1	6
Prostate	2	0
Rectal	1	4

**Table 2:** Common themes for declining or not completing optional MRI scans, including quotes.

Themes and quotes for declining / not completing optional MRI scans	
Theme	Supporting quotes
MRI is too uncomfortable (claustrophobia, scan duration)	“MRIs are very hard for me. I need to be drugged to do it. I am extremely claustrophobic in a way that makes me feel physically unwell. I would vomit, get dizzy, and have double vision. I can’t sit still- I need to be prescribed Xanax. - *Study 2 Participant 57*
Generally feeling overwhelmed	“My chemotherapy schedule was already too much. I could not handle another thing. I did not need it. I was going through so much already with my infusions. Besides, I didn’t see the benefit [to me] of doing it. I only heard the time commitment. The MRI process itself is also not comfortable or appealing” - *Study 3 Participant 18*
Scheduling (not enough time, cannot find an appropriate time)	“I was originally, and still am, willing to do the MRI. But I am unwilling to come in on another day other than when I must go to the hospital for my infusions because I live 45 minutes away. It is time and distance, and the MRI was not available at the time I was at the hospital already, so I didn’t do it.” - *Study 3 Participant 17*
No personal health benefit	“The MRI was an additional thing. It didn’t have any benefit to me. I didn’t understand the purpose and why I was doing it. I am not feeling any neuropathy, so why would I do an MRI? I was open initially, but once I thought more about it [I] decided to say no. It was a scheduling issue too.” - *Study 3 Participant 14* “...I need to understand the benefit to me as a participant. It’s one thing if it has medical benefits or at least perceived benefit from a research standpoint.” - *Study 3 Participant 12*

**Table 3:** Common themes for completing optional MRI scans, including quotes.

Themes and quotes for completing optional MRI scans	
Theme	Supporting quotes
Wanting to help future patients and contributing to research	“I initially said yes, but it became a scheduling issue on my end and on the MRI team. But I said yes because I saw it as benefiting the study and therefore it will help other people in the future.” - *Study 3 Participant 20* “I decided to do the MRI because it was beneficial to the research itself and I wanted to help. I was curious what the images would show and what that would mean. I don’t know why the MRI is a part of this research, but if I did I would be even more motivated to participate.” - *Study 2 Participant 58*
General curiosity	“I did it just to find out more information and see if it helps the study learn more about neuropathy.” - *Study 3 Participant 18* “I have never had an MRI before. I am not a claustrophobic person, unless it takes too long, and wanted to have experience with an mRi before having "a real one." [the study MRI] was quieter than some of the others I have since had.” - *Study 3 Participant 7* “I am willing to participate in any research or part of research. I am not claustrophobic and there is no downside for me really. I am curious about the outcome of the study and the findings it will yield.” - *Study 2 Participant 58*
Mistakenly thought to be for patienťs benefit	“I thought it was a part of the process and that it would make me well. I saw that the MRI was for MY benefit- something to help me. People who said no should not if they have never researched or tried it. It could be something that helps them. If they don’t do it, its because they don’t see how it can help them.” - *Study 3 Participant 15* “[I don’t mind MRI], its always good to do it to make sure you don’t have anything funky. I wish that we got them more regularly to just find stuff like this cancer, which I otherwise would have never known about.” - *Study 3 Participant 19*
Compensation	“I decided to do it because it seemed to make for a more complete participation in the study. It was also compensated. If it hadn’t been compensated, then I probably would not have done it.” - *Study 2 Participant 56*

**Table 4:** Common barriers faced by patients participating in magnetic resonance imaging (MRI) research studies and possible solutions to improve study participation in brain imaging research in cancer.

Barriers to patients	Possible solutions
Patient has anxiety	Clear communication of the instructions, what to expect before, during, and after the scan including scan duration, noise, and constricting environment, and temperature. Providing encouragement and progress updates during the scan session using the microphone/speaker system built into the scanner. Provide strategies to manage anxiety (distraction, focus on breathing when possible), using calming stimuli before and during the scan (music, pictures, videos) when possible. Using alternative, portable brain imaging systems such as fNIRS or EEG that do not restrict participant movement or put them in an enclosed space.
Patient experiences discomfort from lying down or staying still for a long duration	Option to shorten scan time by collecting the higher priority scans first, giving the option to get out of the scanner, earlier if needed. Using alternative, portable brain imaging systems such as fNIRS or EEG that are more tolerant to movement and allow flexibility in terms of supine, prone or upright patient positions. Portable imaging techniques also provide the option to perform at-home assessments, improving accessibility to brain imaging research.
MRI scanner schedule is busy	Use a waitlist to see if scan cancelations open a desirable time. Try to schedule scans far in advance (e.g., if a study has two scans 6 weeks apart, schedule the second scan immediately after consent). Use a shorter scan protocol that fits into a shorter scan time to at least collect some data instead of nothing at all. Using alternative, portable brain imaging systems such as fNIRS or EEG that can be solely owned by the research group and can be brought to the patient in their home, clinic, infusion, or a mutually convenient location. The development of compact, light-weight versions of these techniques allow large scale clinical trials involving a diverse group of patients from community-based or multi-site research.
Cannot travel to an MRI scanner (e.g., due to distance, time, or cost)	Providing patients with transportation (Lyft, Uber) Simpler scanning protocol that can be acquired at an MRI scanner closer to the participant (even if the data quality is reduced or if the scanner is of lower performance) Using alternative, portable brain imaging systems such as fNIRS or EEG that can be brought to the patient in their home, clinic, chemotherapy infusion, or a mutually convenient location.
Patient has MRI contraindications	During study design, consider the likelihood of the patients having MRI contraindications (e.g., if their treatments involve implanting objects that are not MRI-safe, e.g., chest expanders, cardiac stents) Using alternative, portable brain imaging systems such as fNIRS or EEG that are non-invasive, does not involve radiation, and allow long durations of safe monitoring.
Low research literacy and mistrust of research	Involve patient advocates to help educate patients about research procedures and their rights as participants Explain what the images are used for and why alternative methods are insufficient Explain how prior studies have established the current standard of care, with specific examples
Inaccurate understanding of the study expectations and outcomes	If possible, use infographics to explain the study design more simply, making sure to include scan-associated benefits/risks

fNIRS - functional Near Infra-Red Spectroscopy.
